# Do postponed dental visits for financial reasons reduce quality of life? Evidence from the Survey of Health, Ageing and Retirement in Europe

**DOI:** 10.1007/s40520-020-01536-w

**Published:** 2020-04-09

**Authors:** Richelle Valdez, Ghazal Aarabi, Kristin Spinler, Carolin Walther, Christopher Kofahl, Elzbieta Buczak-Stec, Guido Heydecke, Hans-Helmut König, André Hajek

**Affiliations:** 1 Department of Prosthetic Dentistry, Center for Dental and Oral Medicine, University Medical Center Hamburg-Eppendorf, Hamburg, Germany; 2 Institute of Medical Sociology, Center for Psychosocial Medicine, University Medical Center Hamburg-Eppendorf, Hamburg, Germany; 3 Department of Health Economics and Health Services Research, Hamburg Center for Health Economics, University Medical Center Hamburg-Eppendorf, Hamburg, Germany

**Keywords:** Dental visits, Dental care, Dental health services, Health services accessibility, Dental avoidance, Quality of life

## Abstract

**Background:**

There is a lack of studies investigating the impact of postponed dental visits due to financial constraints on quality of life.

**Aims:**

The aim of this study was to identify whether these factors are associated longitudinally.

**Methods:**

Data were derived from waves 5 and 6 of the “Survey of Health Ageing and Retirement in Europe” (SHARE). The analysis focused on Germany (*n* = 7506). The widely used CASP-12 was used to quantify the quality of life. Postponed dental visits for financial reasons in the preceding 12 months (no, yes) were used as the main explanatory variable. Socioeconomic and health-related covariates were included in regression analysis.

**Results:**

Gender stratified regression analysis showed that quality of life decreased with the presence of postponed dental visits due to financial reasons in men. Furthermore, quality of life decreased with the worsening of self-rated health in both men and women. The outcome measure was not associated with age, marital status, income, and chronic diseases in both sexes.

**Discussion:**

Study findings suggest that postponing dental visits due to financial constraints contributes to a decreased quality of life among older men.

**Conclusion:**

Efforts to avoid these circumstances might help to maintain the quality of life in older men.

## Introduction

Interest in the assessment of health-related quality of life (HrQoL) in medicine has grown considerably over the past 30 years [1]. The reason for this may include its manifold applicability in various capacities, such as in facilitating treatment decisions [1], evaluation of treatment [2], shaping health policy [1], and determining the burden of disease and disability [2]. It is also speculated that knowledge of the quality of life from patients in clinical settings could improve patient-physician communication by providing an opportunity to identify problems and preferences [3].

Hundreds of HrQoL instruments have been developed [4], which can be classified either as generic or specific [1, 4]. Generic instruments are designed to measure HrQoL across all diseases, populations, and medical interventions (e.g. CASP-12) [1, 4]. Often used generic HrQoL include the Medical Outcomes Study 36-Item Short Form (SF-36) Health Survey and the EuroQoL Instrument (EQ-5D) [4]. Specific HrQoL target a particular disease/problem, population, or function/dysfunction [1, 4]. An example of a specific HrQoL instrument is the Oral Health Impact Profile (OHIP), which measures respondents’ perception of the impact of oral disease on their well-being [5, 6].

HrQoL has also become an outcome of interest in dentistry, where it has not only been used to quantify and evaluate the impact of oral treatments [7–9] and oral health conditions [10–12], but also to predict dental service utilization patterns [13]. It has been shown that problem-oriented dental attendance was significantly associated with low oral HrQoL (i.e. high functional limitation, pain, etc.) in comparison to those who visit the dentist for routine care [13]. Dental visits were also observed in an Australian study to be associated with the improvement of oral HrQoL over time [14]. Contrary to these results, another study did not observe an association between dental care attendance and oral HrQoL, depending on residential location [15].

Despite the research done about HrQoL and dental service utilization patterns, little is known about the relationship between postponement dental visits due to financial costs and HrQoL. Exploring this relationship can on the one hand provide relevant information for determining access barriers to dental health care, which could decrease dental health outcomes due to non-attendance and on the other hand help to understand how HrQoL is influenced by postponed dental visits. To bridge this gap in knowledge, the present study has investigated whether the postponement of dental visits due to costs has an impact on HrQoL.

## Methods

### Sample

For the current study, we used data from two waves (wave 5 in 2013 and wave 6 in 2015) of the Survey of Health Ageing and Retirement in Europe (SHARE) study [16]. We restricted our analyses to Germany. For reasons of data availability, previous waves were excluded.

In European countries and Israel, non-institutionalized individuals aged 50 and above (and their spouses) took part in the SHARE study covering topics such as health, social networks and socioeconomic status. The SHARE study includes representative samples of the population. Paper and pencil, as well as computer-assisted personal interviews, were conducted. Börsch-Supan and colleagues provide further details of the SHARE study [17].

During waves 1 to 4, SHARE has been reviewed and approved by the Ethics Committee of the University of Mannheim several times. Wave 4 of SHARE and the continuation of the project have been reviewed and approved by the Ethics Council of the Max-Planck-Society (most recently in 2018). Prior to the CAPI interview, oral consent was given. This consent procedure was approved by the Ethics Committees. The Ethical Commission agreed that verbal consent is sufficient and that written consent statements are not necessary to conduct the SHARE interviews.

### Outcome measure: quality of life

Quality of life was measured using the CASP-12 [18]. On a 4-point scale from 1 to 4, individuals rated their feelings and situations (from “never” to “often”). The total score of these 12 items ranges from 12 to 48 (higher values correspond to a better quality of life).

It has been shown that CASP-12 is a valid and reliable tool for assessing the quality of life in old age [19]. This has also been confirmed in very recent years [20, 21]. The CASP-12 is highly correlated with the CASP-19 and the Life Satisfaction Index [22]. The CASP-19 and the CASP-12 were also used in other large cohort studies.

### Independent variables

The key independent variable was quantified as follows: “In the last twelve months, to help you keep your living costs down, have you postponed visits to the dentist?” (no, yes). In regression analysis, it was also adjusted for age, family status (married and living together with spouse; registered partnership; married, living separated from spouse; never married; divorced; widowed), and income. Furthermore, morbidity (based on a count score covering, for example heart attack; high blood pressure or hypertension; high blood cholesterol; stroke or cerebral vascular disease) and self-rated health (from 1 = excellent to 5 = poor) were used.

### Statistical analysis

In a landmark study, Ferrer-i-Carbonell and Frijters [23] demonstrated that it is of great importance to control for unobserved heterogeneity in well-being research. Hence, the link between the postponement of dental visits and quality of life was analyzed using linear fixed effects (FE) regressions. Unlike other widely used regression techniques, FE regression also provides consistent estimates when unobserved factors such as genetic disposition exist that are associated with independent variables [24].

Only changes within individuals over time are used in FE regressions (e.g., changes in income within individuals over time). Therefore, solely factors that can change over time can be included in FE regressions. Cluster-robust standard errors were calculated [25].

## Results

### Sample characteristics

Sample characteristics for 7506 observations included in linear FE regressions stratified by sex are described in Table 1. The average age was 66.2 years (± 9.6 years) in men, and in women, it was 65.1 years (± 9.9). The average quality of life score was 39.2 (± 5.5) in men, and in women, it was 38.9 (± 5.6).

**Table 1:** Characteristics of observations included in fixed effects regression analysis stratified by sex (*n* = 7506; waves 5 and 6, Germany)

	Male (*n* = 3563)		Female (*n* = 3943)	
	*n*/mean	%/(SD)	*n*/mean	%/(SD)
Age in years: mean (SD)	66.2	(9.6)	65.1	(9.9)
Marital status: married and living together with spouse; registered partnership: *N* (%)	2606	73.1%	2574	65.3%
Household net income (per year) in Euro: mean (SD)	33,736.5	(42,315.62)	30,098.3	(39,988.6)
Number of chronic diseases: mean (SD)	1.3	(1.3)	1.1	(1.2)
Self-rated health (1 = “excellent” to 5 = “poor”): mean (SD)	3.2	(1.0)	3.2	(1.0)
Postponed dental visits for financial reasons: no: *N* (%)	3438	96.5%	3766	95.5%
Quality of life (CASP-12): mean (SD)	39.2	(5.5)	38.9	(5.6)

It is worth noting that 182 individuals (77 changes in men, 105 changes women) reported changes in the variable ‘postponed dental visits due to financial reasons’ from waves 5 to 6 in Germany.

### Regression analysis

The results of linear FE regression analysis are depicted in Table 2. Regressions revealed that quality of life decreased with the presence of postponed dental visits due to financial reasons (*β* =  − 1.36, *p* < 0.05) and worsening self-rated health (*β* =  − 0.56, *p* < 0.001) in men. Whereas in women, only a decrease in quality of life was observed in the presence of worsening self-rated health (*β* =  − 0.85, *p* < 0.001). Quality of life was not associated with age, marital status, income, and chronic diseases in both sexes.

**Table 2:** Determinants of quality of life stratified by sex. Results of linear fixed effects regressions (waves 5 and 6, Germany)

Independent variables	Quality of life-men	Quality of life-women
Postponed dental visits for financial reasons (reference category: not having postponed dental visits for financial reasons)	− 1.360* (0.574)	− 0.298 (0.502)
Age	− 0.0745 (0.0569)	0.0233 (0.0565)
Marital status: married and living together with spouse or registered partnership [reference category: others married (living separated from spouse, never married, divorced, widowed)]	− 1.536 (1.070)	− 0.337 (0.780)
Log household net income	0.143 (0.136)	− 0.0995 (0.113)
Self-rated health (1 = “excellent” to 5 = “poor”)	− 0.557*** (0.144)	− 0.847*** (0.163)
Number of chronic diseases	− 0.216 (0.136)	0.0575 (0.126)
Constant	45.92*** (4.276)	41.26*** (4.079)
Observations	3563	3943
Number of individuals	2385	2649
*R*^2^	0.028	0.028

Unstandardized *β*-coefficients were reported; cluster-robust standard errors in parentheses

***p < 0.001, **p < 0.01, *p < 0.05, ^+^*p* < 0.10

## Discussion

### Main findings

The objective of our study was to determine whether postponed dental visits due to financial constraints were associated with quality of life in Germany longitudinally. Gender stratified regression analysis showed that quality of life decreased with the presence of postponed dental visits due to financial reasons (men only) and worsening of self-rated health (men and women).

### Previous research and possible explanations

The main result of the present study demonstrated that postponing dental visits due to costs contributes to a decreased quality of life among older men. To our knowledge, little research has been conducted so far that has explored the relationship between postponement of dental visits due to costs and quality of life. Despite this, there have been studies that have investigated dental attendance and oral HrQoL. Previous research observed no significant association between impaired oral HrQoL and dental service use [14, 26], while other studies did see an association with impaired oral HrQoL with problem-oriented dental visits [13, 27] and a protective effect of routine dental visits on oral HrQoL [28]. Like problem-oriented dental visits, postponing dental visits due to cost could be seen as “negative” oral health behaviour that may be associated with impaired quality of life and also oral HrQoL.

Another result worth noting is that postponing dental visits due to costs and quality of life did not show significant results for women. Although no research exists specifically about this topic, this result may be an indication of gender differences of health behaviour and attitudes in dental service utilization behaviours, in which women may be more health-conscious than men. Previous studies have shown that women are more interested in health-related topics and are reported to actively seek health-related information more than men [29, 30].

Not surprisingly, quality of life was also observed to decrease in the presence of worsening self-rated health, regardless of gender. This results are in agreement with other studies that have observed that poor self-rated health is associated with lower quality of life. For example, one study found that those who reported poorer quality of life are significantly more likely to rate their health poorly [31]. Studies using the Nottingham Health Profile (NHP) to measure the quality of life in a variety of patient groups report the highest correlations between self-rated health and the NHP dimensions for pain, mobility, and energy were observed [32, 33]. In this way, this paper adds to the existing evidence that quality of life and self-rated health are highly correlated, and indeed may be interconnected concepts that influence each other, in which construct overlap may exist depending on the individual quality of life measures used. Because self-rated health is an indicator for HrQoL and usually explains the biggest part of the response variation in the goodness of fit, it surely is an essential control parameter in our model. In light of this, it is interesting that postponement of dental visits due to financial reasons remains an independent predictor for a decrease in HrQoL in men.

### Strengths and limitations

The current study adds insight into the link between postponing dental visits for financial reasons and quality of life longitudinally. Using FE regressions, the challenge of unobserved heterogeneity was mitigated [34]. In this study, a nationally representative sample was used, as well as the well-known and validated CASP-12. However, future validation studies are of interest. A small attrition bias as well as a small sample selection bias cannot be dismissed in SHARE data [35].

Another possible weakness of this study is that no clinical data was collected which may have given concreter insights on how health and perception of one's quality of life may affect patients’ dental service utilization behaviour. Although CASP-12 is a well-validated instrument to measure the quality of life, data derived from an oral health-related quality of life instrument may have been a better choice, given the topic of this paper. Previous research comparing generic quality of life instruments and specific quality of life instruments has provided evidence that (condition) specific quality of life instruments may be more sensitive in detecting changes in quality of life over time than the generic quality of life instruments [36].

## Conclusions

Postponing dental visits due to financial constraints contributes to a decreased quality of life among older men. This may suggest that emphasizing the importance of maintaining good oral health (i.e. visiting the dentists regularly) and making dental care more accessible could not only improve oral health outcomes but also maintain or improve quality of life in men.
